# Virological failure and development of new resistance mutations according to CD4 count at combination antiretroviral therapy initiation

**DOI:** 10.1111/hiv.12302

**Published:** 2015-08-25

**Authors:** S Jose, K Quinn, D Dunn, A Cox, C Sabin, S Fidler, the UK CHIC and UK HDRD Steering Committees, Jonathan Ainsworth, Sris Allan, Jane Anderson, Abdel Babiker, David Chadwick, Valerie Delpech, Martin Fisher, Brian Gazzard, Richard Gilson, Mark Gompels, Phillip Hay, Teresa Hill, Margaret Johnson, Stephen Kegg, Clifford Leen, Fabiola Martin, Mark Nelson, Chloe Orkin, Adrian Palfreeman, Andrew Phillips, Deenan Pillay, Frank Post, Jillian Pritchard, Memory Sachikonye, Achim Schwenk, Anjum Tariq, John Walsh, Alicia Thornton, Adam Glabay, N Perry, S Tilbury, E Youssef, D Churchill, R Everett, D Asboe, S Mandalia, H Korat, C Taylor, Z Gleisner, F Ibrahim, L Campbell, N Brima, I Williams, M Youle, F Lampe, C Smith, R Tsintas, C Chaloner, S Hutchinson, S Huntington, N Mackie, A Winston, J Weber, F Ramzan, M Carder, J Lynch, J Hand, C Souza, S Munshi, S Miller, C Wood, A Wilson, S Morris, S Allan, A Palfreeman, K Memon, A ewszuk, D Chadwick, E Cope, J Gibson, S Kegg, P Main, Dr Mitchell, Dr Hunter, P Hay, M Dhillon, F Martin, S Russell‐Sharpe, A Harte, S Clay, A Tariq, H Spencer, R Jones, C Atkinson, V Delpech, M Sachikonye, Celia Aitken, Anton Pozniak, Patricia Cane, Duncan Clark, Simon Collins, Samuel Douthwaite, Ellen White, Christophe Fraser, Anna Maria Geretti, Antony Hale, Stéphane Hué, Steve Kaye, Paul Kellam, Linda Lazarus, Andrew Leigh‐Brown, Tamyo Mbisa, Samuel Moses, Eleni Nastouli, Erasmus Smit, Kate Templeton, Peter Tilston, Daniel Webster, Hongyi Zhang, Jane Greatorex, Jane Mullen, Richard Tandy, Tracy Fawcett, Mark Hopkins, Lynn Ashton, Claire Booth, Ana Garcia‐Diaz, Jill Shepherd, Matthias L Schmid, Brendan Payne, Spiro Pere, Jonathan Hubb, Stuart Kirk, Rory Gunson, Amanda Bradley‐Stewart

**Affiliations:** ^1^ Research Department of Infection and Population HealthUCLLondonUK; ^2^ Department of MedicineImperial College LondonLondonUK; ^3^ Medical Research Council Clinical Trials Unit at UCLLondonUK; ^4^ Infection and ImmunityImperial College Healthcare NHS TrustLondonUK

**Keywords:** antiretroviral therapy, CD4 count, HIV resistance, virological failure

## Abstract

**Objectives:**

No randomized controlled trials have yet reported an individual patient benefit of initiating combination antiretroviral therapy (cART) at CD4 counts > 350 cells/μL. It is hypothesized that earlier initiation of cART in asymptomatic and otherwise healthy individuals may lead to poorer adherence and subsequently higher rates of resistance development.

**Methods:**

In a large cohort of HIV‐positive individuals, we investigated the emergence of new resistance mutations upon virological treatment failure according to the CD4 count at the initiation of cART.

**Results:**

Of 7918 included individuals, 6514 (82.3%), 996 (12.6%) and 408 (5.2%) started cART with a CD4 count ≤ 350, 351–499 and ≥ 500 cells/μL, respectively. Virological rebound occurred while on cART in 488 (7.5%), 46 (4.6%) and 30 (7.4%) with a baseline CD4 count ≤ 350, 351–499 and ≥ 500 cells/μL, respectively. Only four (13.0%) individuals with a baseline CD4 count > 350 cells/μL in receipt of a resistance test at viral load rebound were found to have developed new resistance mutations. This compared to 107 (41.2%) of those with virological failure who had initiated cART with a CD4 count < 350 cells/μL.

**Conclusions:**

We found no evidence of increased rates of resistance development when cART was initiated at CD4 counts above 350 cells/μL.

## Introduction

Although morbidity and mortality benefits of starting combination antiretroviral therapy (cART) at CD4 counts > 350 cells/μL have been reported in cohort studies 1, 2, there is little randomized evidence on the individual risk–benefit ratio of initiating combination antiretroviral therapy (cART) at higher CD4 counts 3. The randomized controlled Strategic Timing of AntiRetroviral Treatment (START) trial has recently investigated the optimal timing of cART initiation in order to improve morbidity and mortality outcomes in HIV‐positive individuals 4. Nevertheless, there have already been changes to national and international HIV treatment guidelines 5, 6, largely driven by the impact of cART on viral transmission 7 and a pragmatic approach to cART roll out programmes.

As adherence to cART has been associated with perceived “need” for treatment 8, there is concern that a recommendation to start cART at higher CD4 counts may be met with patients’ ambivalence to cART, leading to suboptimal adherence and antiretroviral resistance. However, there are no data reported in support of this hypothesis to date 9, 10. The START trial will investigate antiretroviral resistance development as a secondary endpoint, and will report these findings after 2016.

Previously, we reported on laboratory‐defined adverse events (LDAEs) according to the CD4 count at initiation of cART 11. We now describe rates of new antiretroviral resistance mutations in those experiencing virological failure, according to the CD4 count at treatment initiation.

## Methods

The UK Collaborative HIV Cohort (CHIC) Study collates routinely collected clinical data on HIV‐positive individuals accessing care across several centres in the UK. The study was approved by a multicentre research ethics committee and by local ethics committees and does not require informed consent. Similarly, the UK HIV Drug Resistance Database (HDRD) collates results of routine resistance testing of HIV‐positive individuals accessing care. A combined UK CHIC – UK HDRD dataset was used for analysis. Individuals were included who initiated cART (≥ 3 antiretroviral drugs) between 2000 and 2011 with a baseline CD4 count and resistance test result available, and who achieved an undetectable viral load on cART. Pregnant women were excluded.

Virological rebound was defined at the first occurrence of two consecutive viral loads > 400 HIV‐1 RNA copies/mL following an undetectable viral load. Virological rebound occurring while on cART was classed as treatment failure and evaluated for resistance development. Virological rebound that occurred immediately following a treatment discontinuation was not evaluated for resistance development. The earliest resistance test result available up to 1 month before or 6 months after the date of virological failure was used. We defined new resistance as the presence of any new major resistance mutation 12 not present in the baseline genotype, stratifying results according to the CD4 count at cART initiation (≤ 350, 351–499 and ≥ 500 cells/μL).

## Results

In total, 9288 people had initiated cART since 2000 and had a baseline CD4 count and resistance test result available. Of these, 8445 (91%) achieved an undetectable viral load. A further 527 were excluded due to pregnancy. Of 7918 included, 6514 (82.3%), 996 (12.6%) and 408 (5.2%) started cART with a CD4 count ≤ 350, 351–99 and ≥ 500 cells/μL, respectively.

Those with a baseline CD4 count 351–499 or ≥ 500 cells/μL were more likely to be men who have sex with men (MSM), of white ethnicity, co‐infected with HCV and having started cART in a later calendar year than those with a baseline CD4 count ≤ 350 cells/μL (*P *< 0.0001). Those with a CD4 count ≥ 500 cells/μL were more likely to start a ritonavir‐boosted protease inhibitor (PI/r)‐based regimen (43.9 *vs*. 29.3% 351–499 cells/μL and 25.8% < 350 cells/μL) and less likely to start a nonnucleoside reverse transcriptase inhibitor (NNRTI)‐based regimen (52.0 *vs*. 64.7% and 69.5%, respectively). At baseline, 255 (4.0%), 26 (2.7%) and 23 (5.9%) of those with CD4 count ≤ 350, 351–499 and ≥ 500 cells/μL were not fully susceptible to ≥ 1 drug in their cART regimen (*P* = 0.02).

Virological rebound occurred in 806 (10.2%) individuals, of whom 564 were receiving cART at the time of rebound: 488 (7.5%), 46 (4.6%) and 30 (7.4%) with a baseline CD4 count ≤ 350, 351–499 and ≥ 500 cells/μL, respectively. A higher proportion of individuals with a baseline CD4 count ≤ 350 cells/μL received a resistance test at virological failure (Table 1). By the time of virological failure, 30.0% had changed their cART regimen class, and 38.5, 54.4 and 53.3% were on PI/r‐based, and 45.3, 34.8 and 40.0% were on a NNRTI‐based regimens (≤ 350, 351–499 and ≥ 500 cells/μL, respectively). The median (interquartile range) viral load was 3.9 (3.1, 4.8), 4.3 (3.4, 5.0) and 3.6 (3.4, 4.5) log_10_copies/mL and 18.9, 15.2 and 13.3% had a viral load < 1000 copies/mL (≤ 350, 351–499 and ≥ 500 cells/μL, respectively). Only four of 30 with a baseline CD4 count > 350 cells/μL had evidence of new resistance mutations at viral load rebound. All mutations conferred resistance to either the nucleoside reverse transcriptase inhibitor (NRTI) or NNRTI class of antiretroviral drugs. New PI, NNRTI and NRTI resistance mutations were found in 3.3, 26.5 and 27.3% of those with a baseline CD4 count ≤ 350 cells/μL and 18.1% had resistance to more than one class of antiretroviral drug.

**Table 1:** Virological failure and development of resistance according to CD4 count at combination antiretroviral theraphy (cART) initiation

Baseline CD4 count, cells/μL	*N*	Virological rebound defined, *n* (%)	Virological rebound occurring on treatment, *n* (%)	Resistance test, *n* (%)	≥ 1 new resistance mutation, *n* (%)	PI resistance, *n* (%)	NNRTI resistance, *n* (%)	NRTI resistance, *n* (%)
≤ 350	6514	643 (9.9)	488 (7.5)	260 (53.3)	107 (41.2)	8 (3.1)	69 (26.5)	71 (27.3)
351–499	996	88 (8.8)	46 (4.6)	20 (43.5)	3 (15.0)	0 (0.0)	3 (15.0)	2 (10.0)
≥ 500	408	75 (18.4)	30 (7.4)	10 (33.3)	1 (10.0)	0 (0.0)	1 (10.0)	1 (10.0)
Total	7918	806 (10.2)	564 (7.1)	290 (51.4)	111 (38.3)	8 (1.4)	73 (25.2)	74 (25.5)
*P*‐value^a^	< 0.0001		0.005	0.056	0.012			

PI, protease inhibitor; NNRTI, non‐nucleoside reverse transcriptase inhibitor, NRTI, nucleoside reverse transcriptase inhibitor.

940 (11.9%) people had at least one major resistance mutation at baseline; of these, 778 (11.9%), 113 (11.4%) and 49 (12.0%) had baseline CD4 count ≤ 350, 351–499 and ≥ 500 cells/μL (*P *= 0.86).

a All *P*‐values based on a chi squared test.

## Discussion

Our results do not demonstrate an increased risk of virological failure on cART when therapy is initiated at CD4 counts > 350 cells/μL. In fact, there appeared to be a reduced risk of developing a major resistance mutation when cART was initiated at CD4 counts > 350 cells/μL. Greater use of PI/r‐based regimens with a higher genetic barrier to resistance in this group may explain the effect of seeing fewer resistance mutations emerge when virological failure occurred 13. However, our findings are consistent with other studies 9, 10.

Higher rates of virological rebound were observed in the group that started cART at CD4 counts above 500 cells/μL. The majority of rebounds in this group occurred following treatment discontinuation, with the proportion of patients experiencing rebound while reportedly still receiving cART being similar across groups. Reasons for treatment discontinuation in this group are unknown; however, it is possible that some of these individuals were enrolled in trials of treatment interruption strategies that were undertaken during this time period 14. However, this trend towards more treatment interruption in those starting cART with a high CD4 count raises some concerns. Depending on the specific drugs included in the regimen as well as the timing of stopping, there is the potential for viral replication to occur in the presence of sub‐optimal levels of cART following treatment discontinuation, thus leading to selection of drug‐resistant virus, particularly for regimens containing NNRTIs 15. Of those who experienced virological rebound following treatment interruption [45 (11%) ≥ 500, 42 (4.2%) 351–499, 155 (2.4%) ≤ 350 cells/μL), nine (20%), nine (21)% and 62 (40%) had a resistance test (> 500, 351–499 and < 350 cells/μL, respectively) with only one (11.1%) person having new resistance mutations detected for CD4 counts 351–499 cells/μL and 15 (24.2%) for CD4 counts ≤ 350 cells/μL.

Fewer resistance tests appeared to be undertaken in individuals experiencing virological failure who had initiated treatment with CD4 counts > 350 cells/μL, raising concerns that resistance mutations may be missed in this group. However, this may be a chance finding and reasons for a lower rate of testing in this group are unclear; lower viral load at virological failure did not appear to explain this and no significant predictors of resistance testing at virological failure were found in any CD4 strata.

Our analyses are limited because reasons for starting cART at high CD4 counts outside current national guidelines are not known. Caucasians and MSM were over‐represented amongst those starting therapy with CD4 counts > 350 cells/μL, indicating that subgroups traditionally presenting to HIV services earlier in the United Kingdom tend to initiate treatment earlier 16. This may indicate an underlying selection bias amongst those starting therapy early, as native English speakers and UK nationals with greater access to healthcare and of potentially higher educational status may opt to start therapy earlier. However, repeating our analysis including only MSM, we saw similar patterns by CD4 count strata, with 35.1, 12.5 and 11.1% of those with CD4 counts < 350, 351–499 and > 500 cells/μL (respectively) having new resistance mutations when tested at virological rebound. Furthermore, in this observational setting, those who have been motivated to start cART at higher CD4 counts may be more likely to have better adherence to treatment and may therefore be less likely to either experience virological rebound or develop resistance to antiretrovirals.

Despite certain limitations, we have not found evidence of an increased risk of resistance development at virological failure amongst people initiating cART at CD4 counts > 350 cells/μL.

## Appendix (1)

### UK CHIC Study

Steering Committee: Jonathan Ainsworth, Sris Allan, Jane Anderson, Abdel Babiker, David Chadwick, Valerie Delpech, David Dunn, Martin Fisher, Brian Gazzard, Richard Gilson, Mark Gompels, Phillip Hay, Teresa Hill, Margaret Johnson, Sophie Jose, Stephen Kegg, Clifford Leen, Fabiola Martin, Mark Nelson, Chloe Orkin, Adrian Palfreeman, Andrew Phillips, Deenan Pillay, Frank Post, Jillian Pritchard, Caroline Sabin, Memory Sachikonye, Achim Schwenk, Anjum Tariq, John Walsh.

Central Co‐ordination: University College London (Teresa Hill, Sophie Jose, Andrew Phillips, Caroline Sabin, Alicia Thornton); Medical Research Council Clinical Trials Unit at UCL (MRC CTU at UCL), London (David Dunn, Adam Glabay).

Participating Centres: Brighton and Sussex University Hospitals NHS Trust (M Fisher, N Perry, S Tilbury, E Youssef, D Churchill); Chelsea and Westminster Hospital NHS Foundation Trust, London (B Gazzard, M Nelson, R Everett, D Asboe, S Mandalia); King's College Hospital NHS Foundation Trust, London (F Post, H Korat, C Taylor, Z Gleisner, F Ibrahim, L Campbell); Mortimer Market Centre, University College London (R Gilson, N Brima, I Williams); Royal Free NHS Foundation Trust/University College London (M Johnson, M Youle, F Lampe, C Smith, R Tsintas, C Chaloner, S Hutchinson, C Sabin, A Phillips, T Hill, S Jose, A Thornton, S Huntington); Imperial College Healthcare NHS Trust, London (J Walsh, N Mackie, A Winston, J Weber, F Ramzan, M Carder); Barts and The London NHS Trust, London (C Orkin, J Lynch, J Hand, C de Souza); Homerton University Hospital NHS Trust, London (J Anderson, S Munshi); North Middlesex University Hospital NHS Trust, London (J Ainsworth, A Schwenk, S Miller, C Wood); The Lothian University Hospitals NHS Trust, Edinburgh (C Leen, A Wilson, S Morris); North Bristol NHS Trust (M Gompels, S Allan); Leicester, University Hospitals of Leicester NHS Trust (A Palfreeman, K Memon, A Lewszuk); Middlesbrough, South Tees Hospitals NHS Foundation Trust (D Chadwick, E Cope, J Gibson); Woolwich, Lewisham and Greenwich NHS Trust (S Kegg, P Main, Dr Mitchell, Dr Hunter), St George's Healthcare NHS Trust (P Hay, M Dhillon); York Teaching Hospital NHS Foundation Trust (F Martin, S Russell‐Sharpe); Coventry, University Hospitals Coventry and Warwickshire NHS Trust (S Allan, A Harte, S Clay); Wolverhampton, The Royal Wolverhampton Hospitals NHS Trust (A Tariq, H Spencer, R Jones); Chertsey, Ashford and St Peter's Hospitals NHS Foundation Trust (J Pritchard, S Cumming, C Atkinson); Public Health England, London (V Delpech); UK Community Advisory Board (M Sachikonye).

### UK HDRD

Steering Committee: Celia Aitken (Gartnavel General Hospital, Glasgow); David Asboe, Anton Pozniak (Chelsea & Westminster Hospital, London); Patricia Cane (Public Health England, Porton Down); David Chadwick (South Tees Hospitals NHS Trust, Middlesbrough); Duncan Churchill (Brighton and Sussex University Hospitals NHS Trust); Duncan Clark (St Bartholomew's and The London NHS Trust); Simon Collins (HIV i‐Base, London); Valerie Delpech (Centre for Infections, Public Health England); Samuel Douthwaite (Guy's and St. Thomas’ NHS Foundation Trust, London); David Dunn, Esther Fearnhill, Kholoud Porter, Anna Tostevin, Ellen White (MRC Clinical Trials Unit at UCL, London)^1^ ; Christophe Fraser (Imperial College London); Anna Maria Geretti (Institute of Infection and Global Health, University of Liverpool); Antony Hale (Leeds Teaching Hospitals NHS Trust); Stéphane Hué (University College London); Steve Kaye (Imperial College, London); Paul Kellam (Wellcome Trust Sanger Institute & University College London Medical School); Linda Lazarus (Expert Advisory Group on AIDS Secretariat, Public Health England); Andrew Leigh‐Brown (University of Edinburgh); Tamyo Mbisa (Virus Reference Department, Public Health England); Nicola Mackie (Imperial NHS Trust, London); Samuel Moses (King's College Hospital, London); Chloe Orkin (St. Bartholomew's Hospital, London); Eleni Nastouli, Deenan Pillay, Andrew Phillips, Caroline Sabin (University College London Medical School. London); Erasmus Smit (Public Health England, Birmingham Heartlands Hospital); Kate Templeton (Royal Infirmary of Edinburgh); Peter Tilston (Manchester Royal Infirmary); Daniel Webster (Royal Free NHS Trust, London); Ian Williams (Mortimer Market Centre, London); Hongyi Zhang (Addenbrooke's Hospital, Cambridge).

Centres contributing data: Clinical Microbiology and Public Health Laboratory, Addenbrooke's Hospital, Cambridge (Jane Greatorex); Guy's and St. Thomas’ NHS Foundation Trust, London (Siobhan O'Shea, Jane Mullen); PHE – Public Health Laboratory, Birmingham Heartlands Hospital, Birmingham (Erasmus Smit); PHE – Virus Reference Department, London (Tamyo Mbisa); Imperial College Health NHS Trust, London (Alison Cox); King's College Hospital, London (Richard Tandy); Medical Microbiology Laboratory, Leeds Teaching Hospitals NHS Trust (Tracy Fawcett); Specialist Virology Centre, Liverpool (Mark Hopkins, Lynn Ashton); Department of Clinical Virology, Manchester Royal Infirmary, Manchester (Peter Tilston); Department of Virology, Royal Free Hospital, London (Claire Booth, Ana Garcia‐Diaz); Edinburgh Specialist Virology Centre, Royal Infirmary of Edinburgh (Jill Shepherd); Department of Infection & Tropical Medicine, Royal Victoria Infirmary, Newcastle (Matthias L Schmid, Brendan Payne); South Tees Hospitals NHS Trust, Middlesbrough (David Chadwick); Department of Virology, St Bartholomew's and The London NHS Trust (Spiro Pereira, Jonathan Hubb); Molecular Diagnostic Unit, Imperial College, London (Steve Kaye); University College London Hospitals (Stuart Kirk); West of Scotland Specialist Virology Laboratory, Gartnavel, Glasgow (Rory Gunson, Amanda Bradley‐Stewart, Celia Aitken).
